# Estimating Rate of Insulin Resistance in Patients with Preeclampsia Using HOMA-IR Index and Comparison with Nonpreeclampsia Pregnant Women

**DOI:** 10.1155/2014/140851

**Published:** 2014-04-09

**Authors:** Farideh Rezaei Abhari, Maryam Ghanbari Andarieh, Asadollah Farokhfar, Soleiman Ahmady

**Affiliations:** ^1^Obstetric Department, Nasibeh Nursing and Midwifery Faculty, Mazandaran University of Medical Sciences, Sari, Iran; ^2^Fatemehzahra Infertility and Reproductive Health Research Center, Babol University of Medical Sciences, Babol, Iran; ^3^Department of Ophthalmology, Mazandaran University of Medical Sciences, Sari, Iran; ^4^Department of Medical Education, School of Medical Education, Shaheed Beheshti University of Medical Science, Tehran, Iran

## Abstract

Women with preeclampsia, independent of obesity and glucose intolerance, exhibit insulin resistance during pregnancy. The purpose of the present study is to determine whether early diagnosis of insulin resistance during pregnancy can predict preeclampsia. Through a case-control study, 675 pregnant women were selected and their first trimester blood was taken. Their fasting blood glucose and insulin were also measured after diagnosis of preeclampsia by 20 weeks of pregnancy. Based on the experiments conducted on 675 women who were 20 weeks past their pregnancy, 375 cases with preeclampsia were selected and assigned to the case group. 35 other pregnant women were put in the control group. Diagnosis criteria for the participants included blood pressure above 140/90 and proteinuria above 300 mg or above +1. Both groups were matched according to age, parity, gestational age, and BMI. Homa-Irand rate of insulin resistance was calculated by HOMA-IR and patients were followed up. Homeostatic model assessments (HOMA-IR) revealed that the average insulin resistance increased during pregnancy among both the case and control groups. There was a significant difference between insulin resistance of these two groups in both first trimester and third trimester and after developing preeclampsia (*P* < 0.001, *P* = 0.021). Insulin-resistance of the group with preeclampsia was higher in first trimester prior to diagnosis as well as the third trimester after diagnosis compared to natural pregnancy under similar conditions. Measurement of insulin resistance in first trimester may be useful in predicting the risk of preeclampsia.

## 1. Introduction

**Figure 1:**
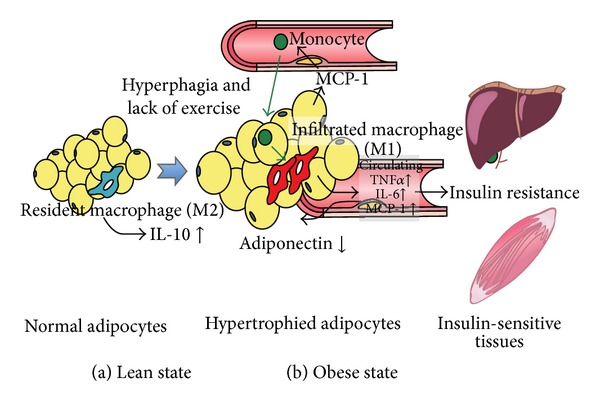
Effect of obesity on insulin resistance.

Insulin is a hormone that facilitates the transport of glucose from the bloodstream into cells. In response to increased blood sugar after a meal, pancreas secretes insulin into the bloodstream. When insulin resistance occurs, the normal amount of secreted insulin is not sufficient in order to deliver glucose into the cells. Pancreas subsequently increases its production of insulin to deliver blood sugar into the cells. Obesity and pregnancy are among the factors which can create insulin resistance. For these conditions there are theories that can explain etiology. Obesity is a cause of insulin resistance in modern societies. Obesity is often accompanied by an increase in fat cell size. This causes changes in adipokines, including a reduction in adiponectin and an increase in tumor necrosis factor alpha and free fatty acids which increase insulin resistance [1] (Figure 1). Many metabolic changes during pregnancy increase adipose tissue and subsequently insulin resistance. Various placental hormones, in addition, alter maternal physiology to supply embryonic requirements. There is also a 30-fold increase in human placental lactogen (hPL) which leads to the secretion of insulin from pancreas [2]. Studies show that hPL plays a role in insulin resistance [3]. 6-fold increase in human chorionic growth hormone is another factor causing insulin resistance [4].

**Figure 2:**
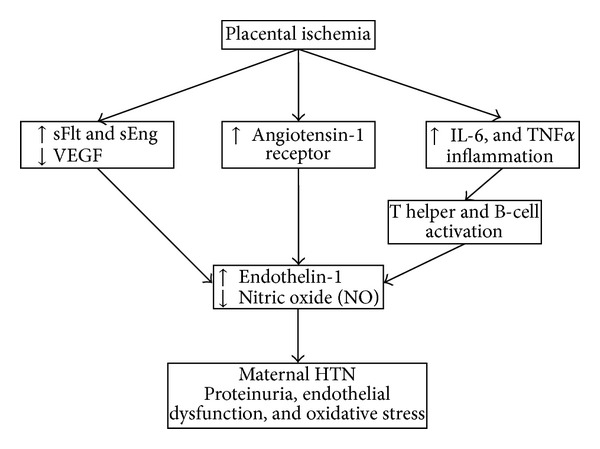
The effect of angiogenic and antiangiogenic factors on endothelial dysfunction.

Preeclampsia is a condition unique to human pregnancy occurring after the twentieth week of pregnancy. Preeclampsia occurs in 2–8% of pregnancies [5, 6] and is associated with maternal and fetal mortality. Preeclampsia is defined as increased systolic blood pressure over 140 mmHg and diastolic blood pressure over 90 mmHg associated with proteinuria. Symptoms can be excessive edema of hands and feet, weight gain over 2 pounds a week, epigastric pain, severe nausea and vomiting, headaches, and vision and brain problems. Preeclampsia risk factors include previous history of preeclampsia, obesity, nulliparity, diabetes mellitus, age over 35 years at first pregnancy, and connective tissue disorders [6]. According to most theories of etiology, preeclampsia refers to maternal abnormal inflammatory response to endothelial damage and hemodynamic instability. Preeclampsia is characterized by placental hypoxia or ischemia, oxidative stress associated with endothelial dysfunction. Recent studies have shown that endothelial dysfunction is induced by antiangiogenic factors which are themselves induced by other factors [6] (Figure 2).

All these changes lead to maternal hypertension and proteinuria which are main criteria for detecting preeclampsia.

Mild preeclampsia is associated with the lowest maternal and neonatal mortality and morbidity rate, while severe preeclampsia before 35 weeks into pregnancy is associated with significant maternal and prenatal complications [7]. Severe preeclampsia occurs when blood pressure reaches over 160/110 and proteinuria is above 5 g in 24-hour urine collection, as shown in Table 1.

In addition to maternal risk factors such as hypertension, type 2 diabetes, antiphospholipid antibody syndrome, obesity, and aging which have been proved to influence preeclampsia, recent studies have attested to the role of genetic factors and immune system in preeclampsia [8]. Reduced uterine-placental blood flow resulting from a combination of hypoxia and imbalances of angiogenic and antiangiogenic factors also exists in preeclampsia [6]. Some other factors in relation to preeclampsia are also under study [9, 10]. Studies show that women with preeclampsia have increased risk for developing diabetes later in life [11]. In another study, blood glucose and insulin levels were measured 2 hours after a 75 g oral glucose use in pregnant women; results showed that people with high blood insulin levels have higher risk for preeclampsia [12]. Additional research in Iran studying the relation between insulin levels and the risk for preeclampsia shows that fasting insulin levels are higher in women with preeclampsia during second trimester before the onset of clinical symptoms compared to normal pregnancy; fasting insulin level considerably increases when disease develops [13]. However, other studies found no relationship between elevated insulin and risk of preeclampsia [14, 15]. Criteria such as multiple pregnancy, prepregnancy fasting blood sugar, prepregnancy hypertension or before 20 weeks into pregnancy, systemic diseases (diabetes, hypertension, etc.), parity, prepregnancy body mass index (BMI), and drug use influence insulin resistance or susceptibility to preeclampsia which were considered in this study. A research on insulin resistance in the second trimester with subsequent preeclampsia claims that insulin resistance in the second trimester of pregnancy is associated with later onset of preeclampsia [16]. There is evidence that the rate of insulin resistance in Asians is different from other races [17]. Considering the serious complications of preeclampsia on the mother and fetus and value of predicting insulin resistance in preeclampsia, the present study suggests a process for the prediction of preeclampsia before the onset of the condition and its prevention.

## 2. Materials and Methods

This case-control study was conducted between September 2010 and September 2011 during which 733 pregnant women who were referred to prenatal care in the time of study were examined during the first trimester. The project was approved by the local ethics committee. These women were selected from 12 clinics in Zanjan which provided prenatal care. The subjects were asked to test fasting blood sugar; their blood pressure was measured; they were examined for multiple pregnancy; they were evaluated for other systemic diseases including renal failure and cardiac ischemia; their BMI was calculated; and finally, they were examined for medication and a family history of diabetes. People with the following criteria were excluded from the study: fasting blood sugar over 95 mg/dL, hypertension over 140/90 mmHg before twenty weeks into pregnancy, history of diabetes, hypertension, and heart and renal disease, BMI over 25 kg/m^2^, and receiving drugs other than routine medications which are used in pregnancy (Table 2).

Finally, 58 women met the exclusion criteria and were excluded from the study; 675 pregnant women did not meet the exclusion criteria and remained in the study. 12-hour fasting blood samples were taken from these 675 pregnant women during the second trimester of pregnancy. The samples were kept in −30°C for next examinations. During the followup, hypertension was measured among the participants who were past 20 weeks into pregnancy. Preeclampsia is defined as a twice-measured hypertension which is over 140/90 mmHg as well as a twice-measured proteinuria rated as above 1+ measured in random urine sample through dipstick or over 300 mg in a 24-hour urine collection (Table 3).

During this period, people were followed up for the risk of preeclampsia. Totally, 35 women were diagnosed with preeclampsia. They were classified in the case group and their 12-hour fasting blood samples were taken. Their fasting insulin and fasting glucose were also measured. The rest of pregnant women with normal blood pressure and previous blood samples were used for control group. The 35 women were assigned to the control group who were matched according to parity, BMI, maternal age, and gestational age after assessing their files in the centre and choosing most similar woman to from healthy group to preeclamptic group (Table 4). 12-hour fasting blood samples were taken from control group to determine blood insulin and glucose.

## 3. Calculation of Insulin Resistance

Insulin levels were measured initially by radioimmunoassay from samples taken from mothers in the second trimester as well as after the twentieth week of pregnancy. Glucose was also measured by glucose oxidase. Insulin resistance was measured by hemostasis model assessment (HOMA-IR) formula [18]. Consider

(1) HOMA-IR  =fasting insulin(μU/mL)×fasting glucose(mg/dL)405.

Data obtained from samples were analysed using the SPSS software, version 17. *t*-test and analysis of variance were used to compare mean values among the two groups of the study. *P* values below 0.05 were considered as significant.

## 4. Results

Of 675 pregnant women, 35 developed preeclampsia. Prevalence of preeclampsia was 5.18 among the population. The patients with preeclampsia were not significantly different from the control group in terms of average age, BMI, parity, and gestational age (Table 5).

**Figure 3:**
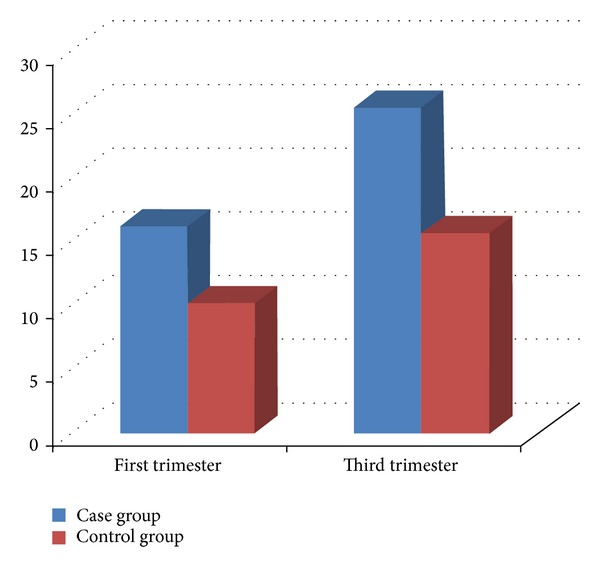
The average fasting insulin level of people with preeclampsia.

Levels of fasting insulin and glucose were substituted into the formula and resistance to insulin was studied among the case and control groups both before and after the development of preeclampsia. The average fasting insulin increased for both case and control groups. Mean score for the level of fasting insulin among people with preeclampsia was higher than that of control group (*P* < 0.01) (Figure 3).

**Figure 4:**
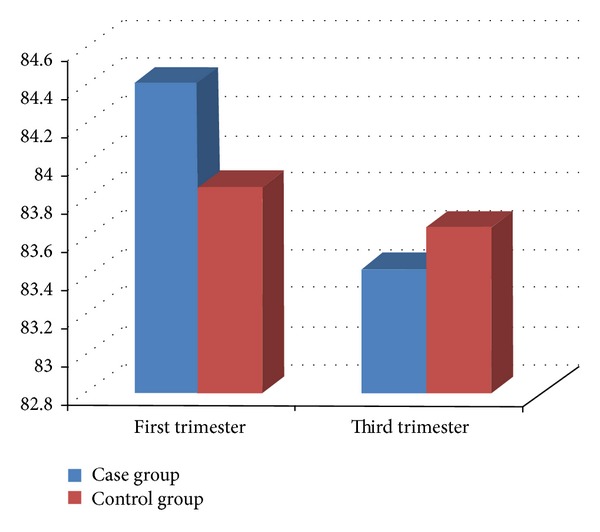
Fasting glucose levels in first and third trimester.

According to results of *t*-test, changes in insulin level were significant for both groups during pregnancy (*P* < 0.01); therefore, insulin rate increased in both groups. No significant difference was found among fasting glucose among the two groups (*P* = 0.724) (Figure 4).

HOMA-IR was calculated for both groups during the first and the third trimesters; in both cases, there was a significant difference between insulin resistances (Table 6).

## 5. Discussion

Having matched case and control groups, this study calculated resistance to insulin using HOMA-IR index and found that the case group differed significantly in levels of insulin resistance both before and after the development of preeclampsia in comparison to the control group. It was thus concluded that insulin resistance could be an important risk factor predicting preeclampsia. Insulin resistance is described as inability of cells to respond to natural function of insulin hormone. Rate of insulin resistance naturally increases during pregnancy. It is believed placenta-derived hormones are the most important factors with the ability to change insulin resistance [19]. The golden standard for direct measurement of insulin resistance is euglycemic glucose clamp test [20]. However, this technique is time consuming and expensive requiring a skilled operator. The present study used indirect HOMA-IR which measures glucose and insulin after 12-hour fasting. Glucose homeostasis depends on its production by liver and insulin secretion from pancreatic beta cells. HOMA-IR shows this dependency in terms of an equation [18]. Our study suggested that insulin resistance during the first trimester among the members of the group which consequently developed preeclampsia was significantly different from the control group. This finding is consistent with previous findings [13, 16]. Other studies did not find such relationship [21]. Using three indices, Parretti et al. measured insulin sensitivity at the early and late stages of pregnancy. These indices were influential in predicting preeclampsia [22]. Using HOMA-IR, Sierra-laguado et al. identified insulin resistance in women with gestational hypertension in early pregnancy [23]; however, this study did not address preeclampsia. Lampinen et al. also found that early preeclampsia influenced later insulin resistance disorder [24]. Kaaja considered insulin resistance as a factor for preeclampsia pathogenesis [25]. According to this evidence, it seems that preeclampsia is associated with increased insulin resistance before the onset of the disease. Most of previous studies suffered problems which influenced the results. Our findings showed that insulin resistance was higher than control group prior to onset of preeclampsia and increased during pregnancy. Little is known on the relationship between insulin resistance and preeclampsia in terms of molecules; however, some studies have been conducted on this regard. Inositol phosphogly can obviously increase by preeclampsia. As the secondary messenger of insulin, this molecule increases metabolic effects of insulin and it is related to insulin resistance [26]. Metabolic disorders causing insulin resistance syndrome can be seen in gestational hypertension disorders including increased levels of plasminogen activator inhibitor −1, leptin, and tumor necrosis factor alpha [27]. Insulin resistance may last for years increasing the likelihood of cardiovascular diseases in these people. These observations suggest that interventions to reduce insulin resistance may reduce increased risk of gestational hypertension and cardiovascular disease.

## Figures and Tables

**Table 1:** Approval criteria for severe preeclampsia.

Persistent hypertension over 160/110 mmHg	
Thrombocytopenia (platelets less than 103 × 100 *μ*L)	
Nephrotic proteinuria (5 g in 24 hr)	
Hemolysis (based on analysis of peripheral blood or increased bilirubin)	
Resistant oliguria (less than 500 cc within 24 hours) (oliguria)	
Liver dysfunction with an unknown cause	
Renal failure (least criteria including increased serum creatinine mg/dL 1 above the base rate)	
Persistent headache	
Persistent pain in right upper quadrant or epigastrium	
The estimated fetal weight below the fifth percentile for gestational age	
Visual scotoma/blurred vision	
Dyspnea with reduced oxygen saturation or pulmonary edema	

**Table 2:** Exclusion criteria.

1	Fasting blood sugar over 95 mg/dL
2	Hypertension over 140/90 mmHg before 20 weeks into pregnancy measured twice
3	History of renal disease, diabetes, hypertension, and heart disease
4	BMI over 25 kg/m^2^
5	Receiving drugs other than routine medications which are used in pregnancy
6	History of diabetes in previous pregnancy

**Table 3:** Diagnosis criteria for preeclampsia.

Systolic hypertension over 140 mmHg or diastolic hypertension over 90 mmHg measured twice in an interval of at least 6 hours after 20 weeks of pregnancy	

300 mg proteinuria in 24-hour urine collection or over 1+ on two random samples during an interval of at least 6 hours or less a week	

**Table 4:** Case and control matching.

	Case	Control
Parity (mod)	2	2
BMI	21/15 kg/m^2^	21/05 kg/m^2^
Maternal age	33/6	32/1
Gestational age	23/2 w	22/8 w

**Table 5:** Characteristics of subjects.

	Control	Preeclampsia	*P* value
BMI (Kg/m^2^)	23.70	23.79	0.788
Number of first pregnancies	23	23	
Age of pregnancy (in weeks)	33.9	34.24	0.922
Average age of mother	27.1	27.61	0.788

**Table 6:** The average HOMA-IR.

	Case group	Control group	*P* value
First trimester	3.09	1.9	0.021
Third trimester	4.76	2.93	0.001

## References

[1] Furukawa N, Araki E (2013). Type 2 diabetes and impaired glucose tolerance. *Nippon rinsho. Japanese Journal of Clinical Medicine*.

[2] Brelje TC, Scharp DW, Lacy PE (1993). Effect of homologous placental lactogens, prolactins, and growth hormones on islet B-cell division and insulin secretion in rat, mouse, and human islets: implication for placental lactogen regulation of islet function during pregnancy. *Endocrinology*.

[3] Beck P, Daughaday WH (1967). Human placental lactogen: studies of its acute metabolic effects and disposition in normal man. *Journal of Clinical Investigation*.

[4] Handwerger S, Freemark M (2000). The roles of placental growth hormone and placental lactogen in the regulation of human fetal growth and development. *Journal of Pediatric Endocrinology and Metabolism*.

[5] Amash A, Huleihel M, Sheiner E, Sapir O, Holcberg G (2007). Preeclampsia as a maternal vascular disease. *Harefuah*.

[6] Eiland E, Nzerue C, Faulkner M (2012). Preeclampsia 2012. *Journal of Pregnancy*.

[7] Sibai BM (2003). Diagnosis and management of gestational hypertension and preeclampsia. *Obstetrics and Gynecology*.

[8] Savaj S, Vaziri N (2012). An overview of recent advances in pathogenesis and diagnosis of preeclampsia. *Iranian Journal of Kidney Diseases*.

[9] Zhang D, Ye D, Chen H (2013). Placental vacuolar ATPase function is a key link between multiple causes of preeclampsia. *ISRN Obstetrics and Gynecology*.

[10] Sowmya S, Ramaiah A, Sunitha T, Nallari P, Jyothy A, Venkateshwari A (2013). Evaluation of Interleukin-10 (G-1082A) promoter polymorphism in preeclampsia. *Journal of Reproduction & Infertility*.

[11] Feig DS, Shah BR, Lipscombe LL (2013). Preeclampsia as a risk factor for diabetes: a population-based cohort study. *PLoS Medicine*.

[12] Romero J, Spinedi E (2013). Two-hour insulinemia after oral glucose overload and women at risk of pregnancy-induced hypertensive disorders. *Hypertension in Pregnancy*.

[13] Malek-Khosravi S, Kaboudi B (2004). Insulin changes in preeclamptic women during pregnancy. *Annals of Saudi Medicine*.

[14] Roberts RN, Traub AL, Kennedy AL, Hadden DR (1998). Glycosylated haemoglobin and hypertension arising in pregnancy. *British Journal of Obstetrics and Gynaecology*.

[15] Roberts RN, Henriksen JE, Hadden DR (1998). Insulin sensitivity in pre-eclampsia. *British Journal of Obstetrics and Gynaecology*.

[16] Hauth J, Clifton R, Roberts J (2011). Maternal insulin resistance and preeclampsia. *American Journal of Obstetrics and Gynecology*.

[17] Misra R, Patel T, Kotha P (2010). Prevalence of diabetes, metabolic syndrome, and cardiovascular risk factors in US Asian Indians: results from a national study. *Journal of Diabetes and its Complications*.

[18] Matthews DR, Hosker JP, Rudenski AS (1985). Homeostasis model assessment: insulin resistance and *β*-cell function from fasting plasma glucose and insulin concentrations in man. *Diabetologia*.

[19] Beck P, Daughaday WH (1967). Human placental lactogen: studies of its acute metabolic effects and disposition in normal man. *Journal of Clinical Investigation*.

[20] DeFronzo RA, Tobin JD, Andres R (1979). Glucose clamp technique: a method for quantifying insulin secretion and resistance. *The American Journal of Physiology*.

[21] Grobman WA, Kazer RR (2001). Serum insulin, insulin-like growth factor-I, and insulin-like growth factor binding protein-1 in women who develop preeclampsia. *Obstetrics and Gynecology*.

[22] Parretti E, Lapolla A, Dalfrà M (2006). Preeclampsia in lean normotensive normotolerant pregnant women can be predicted by simple insulin sensitivity indexes. *Hypertension*.

[23] Sierra-Laguado J, García RG, Celedón J (2007). Determination of insulin resistance using the Homeostatic Model Assessment (HOMA) and its relation with the risk of developing pregnancy-induced hypertension. *American Journal of Hypertension*.

[24] Lampinen KH, Rönnback M, Groop P-H, Kaaja RJ (2008). A relationship between insulin sensitivity and vasodilation in women with a history of preeclamptic pregnancy. *Hypertension*.

[25] Kaaja R (1998). Insulin resistance syndrome in preeclampsia. *Seminars in Reproductive Endocrinology*.

[26] Scioscia M, Gumaa K, Rademacher TW (2009). The link between insulin resistance and preeclampsia: new perspectives. *Journal of Reproductive Immunology*.

[27] Solomon CG, Seely EW (2001). Hypertension in pregnancy a manifestation of the insulin resistance syndrome?. *Hypertension*.

